# Hand Motor Cortex Excitability During Speaking in Persistent Developmental Stuttering

**DOI:** 10.3389/fnhum.2019.00349

**Published:** 2019-10-04

**Authors:** Martin Sommer, Sherko Omer, Alexander Wolff von Gudenberg, Walter Paulus

**Affiliations:** ^1^Department of Clinical Neurophysiology, University of Göttingen, Göttingen, Germany; ^2^PARLO Institute for Research and Training in Speech Therapy, Calden, Germany

**Keywords:** speech motor control, hand motor control, stuttering, motor evoked potentials, transcranial magnetic stimulation

## Abstract

Persistent developmental stuttering (PDS) is a speech fluency disorder characterized by intermittent involuntary breakdowns of speech motor control, possibly related to motor cortex excitability. Whether motor cortex dysfunction extends into hand representations is unclear. We here studied task-dependent modulations of hand motor cortex excitability in 10 right-handed adults who stutter (AWS) and 13 age- and sex-matched fluent speaking control participants (ANS), covering a wide range of tasks in an exploratory study. Before, during and after a null speech/rest task, spontaneous speech, solo reading, chorus reading, singing, and non-verbal orofacial movements, transcranial magnetic stimulation was applied over the primary motor cortex and motor evoked potentials (MEPs) were recorded from the abductor digiti minimi muscle of either hand. In both groups, motor threshold was lower in the left than in the right motor cortex. During task performance, MEP amplitudes increased in both groups. A *post hoc* comparison of spontaneous speech and non-verbal orofacial movements yielded an interaction of group by task with AWS showing larger than ANS MEP amplitude increase in spontaneous speech, but a smaller than ANS MEP amplitude increase in non-verbal orofacial movements. We conclude that hemispheric specialization of hand motor representation is similar for both groups. Spontaneous speech as well as non-verbal orofacial movements are the orofacial tasks that merit further study. The excessive motor cortex facilitation could be reflecting a stronger activation of non-speech muscles during AWS’s speech.

## Introduction

Stuttering is a frequent speech fluency disorder characterized by involuntary disruptions in verbal fluency with audible or silent repetitions or prolongations of sounds or syllables (Bloodstein and Ratner, 2008). It develops in more than 5% of all children without obvious cause (Reilly et al., 2013). Spontaneous recovery is frequent, but stuttering persists in about 1% of adults, predominantly in males (Yairi and Ambrose, 1999). Persistent developmental stuttering has a significant negative impact on quality of life (Koedoot et al., 2011) and socioeconomic success (McAllister et al., 2012).

Shifted laterality with reduced left-hemispheric specialization of speech and non-speech functions is a long-standing theory in stuttering (Travis, 1978). It lead to numerous studies, e.g., on handedness or on dichotic listening, with overall inconclusive results (see overview in chapter four of Bloodstein and Ratner, 2008) Over the past 20 years, functional imaging studies have shown a reduced left hemispheric specialization in adults who stutter (AWS) when looking at speech related brain activity. While fluent speakers (ANS) show a speech related brain activity most prominent in the left hemisphere, adults who stutter produce a speech related brain activity that is excessive and shifted toward the right hemisphere in motor and premotor areas (Brown et al., 2005). Whether this reduced asymmetry is confined to speech motor areas or whether it extends to hand motor areas is less clear. Subtle impairments of bimanual hand motor coordination as shown by others (Vaughn and Webster, 1989; Zelaznik et al., 1997) as well as a right hemispheric shifts of auditory motor integration of hand movements as shown by our group (Neef et al., 2011), in addition to a lack of asymmetry of resting motor threshold of hand motor representations (Sommer et al., 2003), all suggest that the hemispheric asymmetry may not be confined to speech motor areas, but that it may extend to hand motor areas. Our first hypothesis, therefore, was that hemispheric specialization of hand motor representation would be reduced in AWS as compared to ANS.

Given that speaking induces increases in hand motor cortex excitability (Tokimura et al., 1996), and that speech motor preparation is deficient and less left lateralized in AWS as compared to ANS (Neef et al., 2015) our second hypothesis was that lateralization and left hemispheric predominance would be modulated by speaking, i.e., accentuated by speech tasks, attenuated by fluency inducing tasks such as choral speech or singing, and not present in non-verbal as compared to verbal tasks.

## Materials and Methods

The protocol was approved by the University Medical Center Göttingen ethics committee, and we obtained written informed consent before any study related procedure took place.

### Participants

We studied 13 AWS of whom three were excluded from analysis for lack of clear right-handedness (Table 1). The AWS were primarily recruited from an intensive therapy course held in the Kassel Stuttering Therapy Centre, Bad Emstal, Germany. Inclusion was based on the participants’ consent, an absence of medical or self-reported neurological disease, and an absence of medication that influences the excitability of the central nervous system. Given the enrollment in a therapy course and the day-to-day variability of symptom severity, we did not use a minimum percent of syllables stuttered for inclusion in the AWS group. All participants in the AWS group had their diagnosis confirmed by a board-certified phoniatrician prior to enrollment in the therapy program. Sample size was estimated based on previous work using comparable methodologies (e.g., Sommer et al., 2003, 2009). In addition, we recruited 13 fluent speakers matched for age and sex, and carefully selected for the absence of a personal or family history of stuttering or any treatment by a speech-language pathologist. The participants’ speech fluency levels were assessed by a qualified speech-language pathologist who was blinded regarding group status. Stuttering severity was quantified using the German version of the stuttering severity instrument (SSI-3) (Sandrieser and Schneider, 2008), based on two video samples of spontaneous speech as well as reading. Musical practice refers to playing an instrument or singing and was quantified as 1 = never or rarely, 2 = regularly, 3 = professionally. Education was quantified ordinally (1 = school [mittlere Reife], 2 = high school [Abitur], 3 = less than 2 years college, 4 = 2 years college [Bachelor, Vordiplom], 5 = 4 years college [Studium], 6 = postgraduate); handedness was assessed using the Edinburgh Handedness questionnaire (Oldfield, 1971).

**TABLE 1:** Clinical characteristics of participants.

**Measures**	**Stuttering**	**Control**	**Significance**
Participants, *n*	10 (8M, 2F)	13 (10M, 3F)	–
Age in years, mean	28.40 (SD = 8.46)	26.46 (SD = 4.14)	*p* = 0.518 (n.s.)
Handedness, mean	78.28 (SD = 21.72)	73.88 (SD = 18.00)	*p* = 0.370 (n.s.)
Education, mean rank	1.90 (SD = 1.52)	1.85 (SD = 0.38)	*p* = 0.290 (n.s.)
Musical practice, mean	1.20 (SD = 0.42)	1.23 (SD = 0.44)	*p* = 0.860 (n.s.)
Percentage of syllables stuttered, mean	15.17 (SD = 8.42; range, 3.1–30.3)	0.57 (SD = 0.38; range, 0.1–1.3)	*p* < 0.001 (sig.)
SSI-4 mean overall score	31.60 (SD = 7.00)	5.08 (SD = 2.87)	*p* < 0.001 (sig.)
Age of onset	3.90 (SD = 1.10)	–	–

*F, female; M, male; SD, standard deviation. For definition and testing of musical practice and education, see text.*

### Transcranial Magnetic Stimulation

We used a Magstim 200^2^ monophasic TMS device to quantify motor cortex excitability before, during and after the speech tasks. Participants sat in a comfortable reclining chair with armrests, while the experimenter stood behind and out of the participant’s visual field. To detect the optimal motor representation of the abductor digiti minimi muscles of each side, we delivered marginally suprathreshold pulses with a figure-of-eight coil with an outer diameter of 7 cm. The coil was held about 45° posteriolaterally, approximately perpendicular to the presumed location of the central sulcus. We delivered suprathreshold pulses to induce current flow from posterior to anterior in the brain, in order to detect the optimal representation, and then marked the scalp with a pen. We then reduced the stimulus intensity gradually to detect the resting motor threshold (RMT), defined as the minimal intensity where five out of ten consecutive trials elicited contralateral motor evoked potentials (MEP) larger than 50 μV in the target muscle (Rossini et al., 2015). We detected intensity yielding contralateral MEP amplitudes of about 0.5 mV, which were then used as test pulse intensity for the speech tasks (0.5 mV MEPs). These procedures were carried out for each hemisphere separately, in random order. We chose comparatively small baseline MEP amplitudes (Tokimura et al., 1996) because we did not expect any inhibition, so we aimed at increasing the yield with regard to MEP amplitude facilitation by choosing relatively low MEP amplitudes at baseline.

Motor evoked potentials were recorded bilaterally from the abductor digiti minimi muscle using silver-silver chloride cup electrodes in a belly tendon montage with a sampling frequency of 5 kHz, filtered between 20 and 2000 Hz and recorded using a CED 1401 amplifier and Signal 4.16 software (Cambridge Electronic Design, Cambridge, United Kingdom).

### Speech Tasks

In this study, we explored a wide range of six tasks – a rest/null speech task (RE), solo reading (SR), chorus reading (CR), spontaneous speech (SP), singing (SI), and non-verbal orofacial movements (NM), CR and SI being known to be fluency-enhancing in AWS (Bloodstein and Ratner, 2008). Each of the six conditions were presented between a brief period of rest before and after each trial. For the rest condition, the participants were asked to remain silent and not to engage in conversation with the experimenter.

For the two reading tasks, Solo Reading and Chorus Reading, we chose a non-fictional text (annual report by the supervising board of the *Deutsche Bahn*) (Aufsichtsrat der Deutschen Bahn, 2009). This was printed in easily readable font size and displayed on a hold at about 30 cm before the participant’s eyes. Participants were instructed to read either alone (Solo Reading), or loud in chorus with the experimenter standing behind the participant (Chorus Reading). For the Spontaneous Speech task, the text display was removed and participants were encouraged to speak about their recent activities; the experimenter asked open questions to enhance communicative output when necessary. For Singing, participants were encouraged to sing familiar German nursery rhymes [“Alle meine Entchen” (Public domain, 2012a) or “Hänschen klein” (Public domain, 2012b)]. For the Non-verbal Orofacial Movement task, participants were encouraged to produce articulatory movements without verbal content; these included humming, lip smacking, kissing movements, or tongue clicking. To ensure appropriate performance, the different experimental procedures were briefly practiced before starting the actual recordings.

Before each task, we recorded 4 min of suprathreshold TMS pulses at 0.25 Hz, 30 pulses per hemisphere, with the respective test pulse intensity. Details about the forthcoming tasks were only revealed to the participant after this baseline; they were then given four to 5 min to perform that task, i.e., as long as necessary to stimulate either hemisphere 30 times, again at 0.25 Hz. After each task, we recorded another 4 min to obtain 30 MEPs for either side, also with test pulse intensity at 0.25 Hz. An interval of at least 10 min followed before starting the next speech task’s baseline. The order of speech tasks was randomized. The order of hemispheres was stable before, during and after each task, but randomized across tasks. Participants were instructed to refrain from hand and arm movements while performing the tasks.

### Data Analysis

#### Descriptive, Task-Independent Measures

Age and musical practice was compared between groups using unpaired, two-tailed *t*-tests. Education was quantified ordinally and tested non-parametrically using a Mann–Whitney *U*-test, so was handedness. RMT and test pulse intensity were assessed using repeated-measures ANOVAs, each with “group” as between-subjects-factor and “hemisphere of stimulation” (left hemisphere, right hemisphere) as within-subjects factors.

#### Analysis of Raw Amplitudes at Rest, i.e., at Pre-task Baseline

We conducted a repeated-measures ANOVA on the “pre” raw MEP contralateral amplitudes recorded before task onset, with “group” as between-subjects-factor and “speech task” (Rest/null speech task, Solo Reading, Chorus Reading, Spontaneous Speech, Singing, and Non-verbal Orofacial Movements) and “hemisphere of stimulation” (left hemisphere, right hemisphere) as within-subjects factors.

#### Analysis of MEP Amplitudes During Task Performance, Normalized to Pre-task Baseline

Motor evoked potential amplitudes during the speech tasks were normalized to the individual respective baseline and entered in a repeated-measures ANOVA with “group” as between-subjects-factor and “speech task” (Rest/null speech task, Solo Reading, Chorus Reading, Spontaneous Speech, Singing, and Non-verbal Orofacial Movements) and “hemisphere of stimulation” (left hemisphere, right hemisphere) as within-subjects factors.

#### Analysis of MEP Amplitudes After Task Performance, Normalized to Pre-task Baseline

An identical ANOVA was calculated for the MEP amplitudes obtained after the speech tasks, normalized to the individual respective baseline.

In all analyses, the level of significance was set at *p* < 0.05. Statview 5.0 (SAS, Cary, NC, United States) was used for all statistics.

## Results

### Descriptive, Task-Independent Measures

Demographic data of all participants is shown in the Table 1.

**FIGURE 1:**
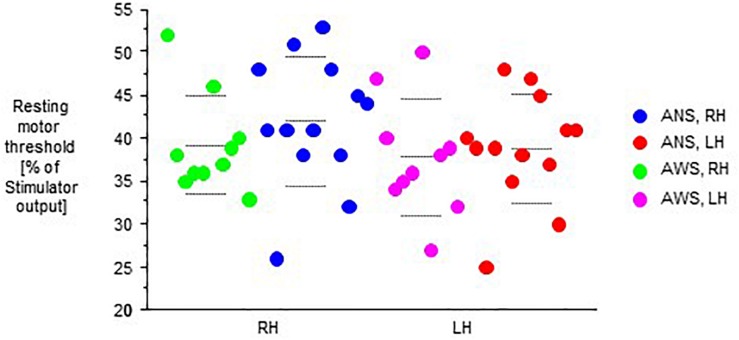
Resting motor threshold as defined in the text, in percent of stimulator output. The **left** (LH) and the **right** (RH) hemisphere were stimulated in random order. Middle dashed line, mean; upper and lower dashed line, single standard deviation.

In both groups, RMT was lower in the left hemisphere than in right hemisphere. The analysis of RMT yielded no main effect of group, but an effect of hemisphere (*F*_(__1__,__21__)_ = 13.98, *p* = 0.001), and no interaction of hemisphere by group (Figure 1). Following the same pattern, stimulus intensity was lower in the left hemisphere than in right hemisphere. It yielded no main effect of group, but an effect of hemisphere (*F*_(__1__,__21__)_ = 9.68, *p* = 0.005), and no interaction of hemisphere by group.

### Raw, Non-normalized MEP Amplitudes

**FIGURE 2:**
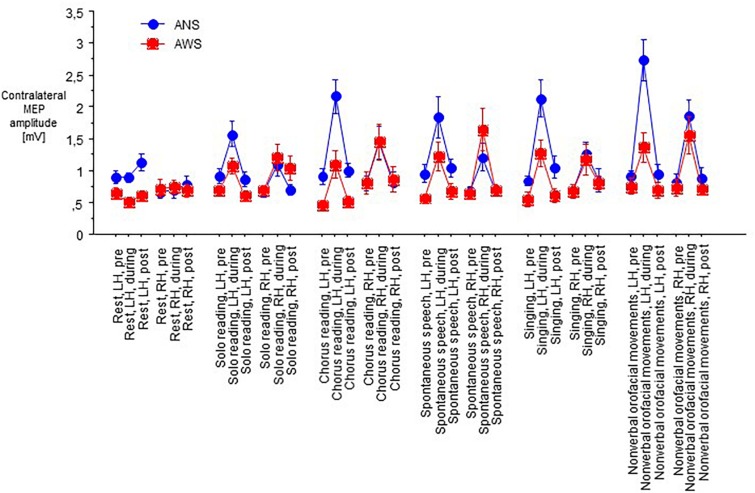
Raw motor evoked potentials (MEPs) from the abductor digiti minimi muscle contralateral to transcranial magnetic stimulation of the motor cortex in 10 adults who stutter and 13 fluent speaking control participants; at baseline (pre), during (D), and after (post) performance of a speech task specified on the abscissa, i.e., rest/null speech task (RE), solo reading (SR), chorus reading (CR), spontaneous speech (SP), singing (SI), or non-verbal orofacial movements (NM). The **left** (LH) and the **right** (RH) hemisphere were stimulated in random order. Mean ± standard error.

Motor evoked potentials amplitudes were small at baseline, increased to a variable extent during task performance, and returned to baseline levels or slightly above after the end of task performance (Figure 2).

**FIGURE 3:**
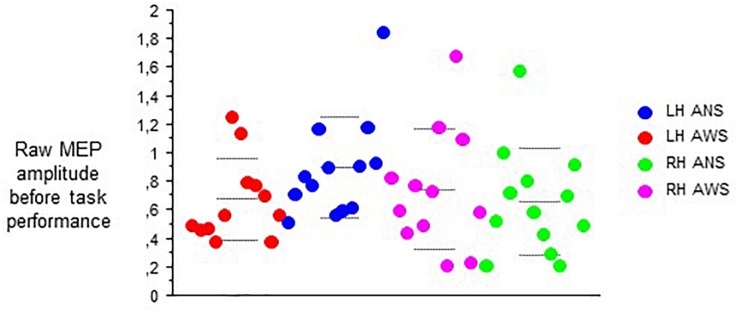
Subset of data from Figure 2; raw MEPs from the abductor digiti minimi muscle contralateral to transcranial magnetic stimulation of the motor cortex recorded at rest before onset of each task. Middle dashed line, mean; upper and lower dashed line, single standard deviation.

At baseline before task onset, the raw contralateral MEP amplitudes did not yield any main effect, but an interaction of hemisphere of stimulation × group (*F*_(__1__,__21__)_ = 10.77, *p* = 0.004, Figure 3), and no other significant interaction. *Post hoc t*-tests showed higher amplitudes in ANS than in AWS for left hemispheric stimulation only. Apparently, a proper between-group matching of MEP amplitudes at baseline had not been successful. The coefficients of variance of these raw MEP amplitudes for the six baselines ranged from 0.30 to 0.54 in the control group and from 0.25 to 0.63 in the patient group. The *F*-tests involving group were all non-significant, excluding major differences in MEP amplitude variability between groups.

### Analysis of MEP Amplitudes Normalized to Pre-task Baseline

**FIGURE 4:**
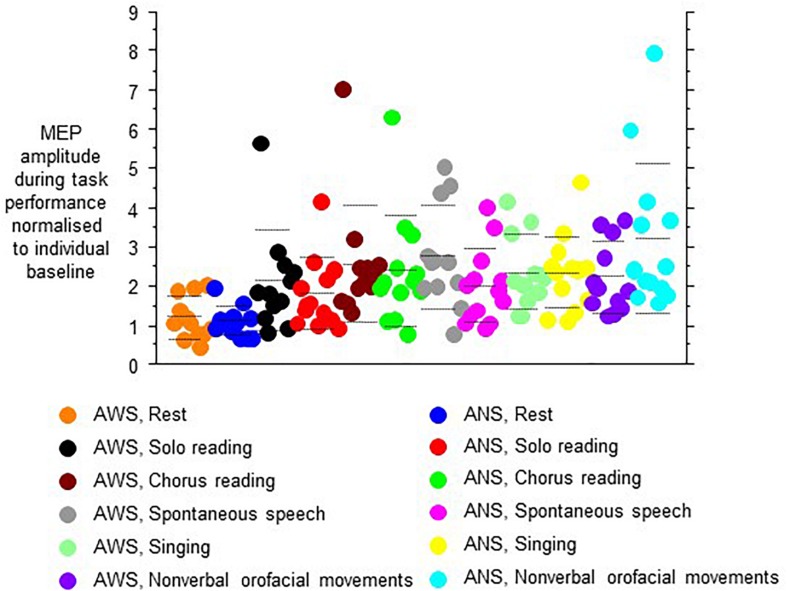
Motor evoked potential amplitudes obtained during task performance, normalized to the respective baseline before task onset. **Left** and **right** hemisphere were stimulated and pooled, data from contralateral recordings are shown. Middle dashed line, mean; upper and lower dashed line, single standard deviation.

The MEP amplitudes during task performance normalized to the individual baseline revealed a main effect of speech task (*F*_(__5__,__105__)_ = 8.13, *p* < 0.0001, Figure 4), and no other main effect or interaction. Specifically, no main effect or interaction involving hemisphere of stimulation was observed, which is why we pooled hemispheres in Figure 4. *Post hoc t*-tests indicated that the Rest/null speech task differed from all other tasks except Solo Reading, and that Solo Reading differed from Non-verbal Orofacial Movements. Compared to ANS, AWS tended to show stronger MEP amplitude increases during Solo Reading, but tended to show smaller MEP amplitude increases during Non-verbal Orofacial Movements; but these between group comparisons failed to reach significance (Figure 4, see also Appendix).

Excluding the Rest/null speech task condition from the above-mentioned analysis did not reveal any effect or interaction involving group.

After the end of task performance, MEP amplitudes returned to baseline values, not yielding any main effects or interactions.

## Discussion

We have investigated hand motor cortex excitability in adults who stutter and a control group before, during and after performing a variety of speech tasks. Our first hypothesis was that hemispheric specialization of hand motor representation would be reduced in AWS as compared to ANS. This was not the case; the left hemisphere yielded a lower motor threshold than the right (Ilic et al., 2004; Davidson and Tremblay, 2013), but this was similar for both groups. In AWS, a lack of asymmetry of motor cortex excitability as compared to ANS has been observed in some (Sommer et al., 2003, 2009), but not in all (Busan et al., 2013) earlier studies. For the hand motor representation, our result does not confirm a long standing lateralization hypothesis with a stronger involvement of the right hemisphere in AWS (Travis, 1978).

Our second hypothesis was that lateralization and left hemispheric predominance would be modulated by speaking, i.e., accentuated by speech tasks, attenuated by fluency inducing tasks such as choral speech or singing, and not present in non-verbal as compared to verbal tasks. More generally, this hypothesis is based on the observation that greater muscular effort usually requires more activation of the motor system, at least within the limits set by central and peripheral fatigue (Mochizuki et al., 2009; Tazoe et al., 2009; Goodwill et al., 2012). The between group differences during speech tasks were more modest than expected, and hemisphere of stimulation did not differentiate the groups under study. Strictly speaking, this hypothesized group-differentiating task-specific activation was not observed, though this conclusion is somewhat hampered by an unexpected bias of baseline MEP amplitudes. AWS differed from ANS by showing some excessive motor cortex facilitation during spontaneous speech, while showing a trend for less motor cortex facilitation during non-verbal orofacial movements. Speculatively, the excessive motor cortex facilitation could be reflecting a stronger activation of non-speech muscles during AWS’s speech (Mulligan et al., 2001). An artifact of prominent hand gestures during speech is unlikely, since we did not observe a prominent extent of hand movements in AWS during task performance. The low motor cortex facilitation required for non-verbal gestures in AWS may pave the way for such these accessory movements in AWS, i.e., the stronger than normal involvement of muscles not immediately required for speech (Wingate, 2002; Mulligan et al., 2003).

Limitations of this study comprise the rather small sample size given the number of conditions tested. This is related (1) to the exploratory nature of task selection, the current study to our knowledge being the first in this patient population, and (2) to the size of the patient population available for study in repeated sessions. Other limitations comprise the recording from a hand muscle, not directly involved in articulation, and putative variations in task performance and effort between groups, which were not overtly present, but not controlled for in a formal manner. The use of fixed intensities rather than input-output curves is another limitation, in particular given the unsuccessful matching of MEP amplitudes.

From this study of a range of speech tasks, we conclude that Spontaneous Speech as well as Non-verbal Orofacial Movements are the orofacial tasks that merit further study, looking at task-related and task-unrelated muscles, and controlling for effort in task performance.

## Data Availability Statement

Pseudonymized data can be accessed by future researchers upon reasonable request based on standard hospital practices.

## Ethics Statement

This study was carried out in accordance with the recommendations of the Ethics Committee of the University Medical Center Göttingen with written informed consent from all subjects. All subjects gave written informed consent in accordance with the Declaration of Helsinki. The protocol was approved by the Ethics Committee of the University Medical Center Göttingen.

## Author Contributions

MS conceived the study, executed the statistical analysis, and wrote the first draft of the manuscript. MS, SO, and AW organized and executed the research project. MS and WP designed the statistical analysis. All authors reviewed and critiqued the statistical analysis and the manuscript.

## Conflict of Interest

The authors declare that the research was conducted in the absence of any commercial or financial relationships that could be construed as a potential conflict of interest.

## Appendix

Given the exploratory task selection in this study, we felt justified to repeat the above-mentioned ANOVA *post hoc*, focusing on the tasks of spontaneous speech and non-verbal orofacial movements. Indeed, this yielded an interaction of task by group (*F*_(__1,21__)_ = 14.82, *p* = 0.032), and no other main effects or interactions.
